# Selection of Soybean Genotypes under Drought and Saline Stress Conditions Using Manhattan Distance and TOPSIS

**DOI:** 10.3390/plants11212827

**Published:** 2022-10-24

**Authors:** Bruno Rodrigues de Oliveira, Alan Mario Zuffo, Jorge González Aguilera, Fábio Steiner, Sheda Méndez Ancca, Luis Angel Paucar Flores, Hebert Hernán Soto Gonzales

**Affiliations:** 1 Pantanal Editora, Nova Xavantina 78690-000, MT, Brazil; 2 Departamento de Agronomia, Universidade Estadual do Maranhão (UEMA), Campus de Balsas, Praça Gonçalves Dias, Balsas 65800-000, MA, Brazil; 3 Departamento de Agronomia, Universidade Estadual de Mato Grosso do Sul (UEMS), Cassilândia 79540-000, MS, Brazil; 4 Escuela Profesional de Ingeniería Pesquera, Universidad Nacional de Moquegua (UNAM), Ilo 18611, Peru; 5 Facultad de Ingeniería de Industrias Alimentarias y Biotecnología, Universidad Nacional de Frontera (UNF), Sullana 20103, Peru; 6 Laboratorio de Biología Molecular y Biotecnología, Escuela Profesional de Ingeniería Ambiental, Universidad Nacional de Moquegua (UNAM), Ilo 18611, Peru

**Keywords:** distance measure, vector space model, abiotic stress, multi-environment, osmotic potential

## Abstract

The search for soybean genotypes more adapted to abiotic stress conditions is essential to boost the development and yield of the crop in Brazil and worldwide. In this research, we propose a new approach using the concept of distance (or similarity) in a vector space that can quantify changes in the morphological traits of soybean seedlings exposed to stressful environments. Thus, this study was conducted to select soybean genotypes exposed to stressful environments (saline or drought) using similarity based on Manhattan distance and the Technique for Order Preference by Similarity to Ideal Solution (TOPSIS) method. TOPSIS is a multi-criteria decision method for selecting the best alternative using the concept of distance. The use of TOPSIS is essential because the genotypes are not absolutely similar in both treatments. That is, just the distance measure is not enough to select the best genotype simultaneously in the two stress environments. Drought and saline stresses were induced by exposing seeds of 70 soybean genotypes to −0.20 MPa iso-osmotic solutions with polyethylene glycol–PEG 6000 (119.6 g L^−1^) or NaCl (2.36 g L^−1^) for 14 days at 25 °C. The germination rate, seedling length, and seedling dry matter were measured. We showed here how the genotypic stability of soybean plants could be quantified by TOPSIS when comparing drought and salinity conditions to a non-stressful environment (control) and how this method can be employed under different conditions. Based on the TOPSIS method, we can select the best soybean genotypes for environments with multiple abiotic stresses. Among the 70 tested soybean genotypes, RK 6813 RR, ST 777 IPRO, RK 7214 IPRO, TMG 2165 IPRO, 5G 830 RR, 98R35 IPRO, 98R31 IPRO, RK 8317 IPRO, CG 7464 RR, and LG 60177 IPRO are the 10 most stable genotypes under drought and saline stress conditions. Owing to high stability and gains with selection verified for these genotypes under salinity and drought conditions, they can be used as genitors in breeding programs to obtain offspring with higher resistance to antibiotic stresses.

## 1. Introduction

Soybean (*Glycine max* (L.) Merrill.) is among the most important crops in the world, constituting one of the largest sources of vegetable oil and animal protein [1,2]. The main producing countries are Brazil, the USA, Argentina, China, and India [3]. Abiotic stresses such as drought and salinity negatively affect world soybean production, constituting limiting factors for soybean cultivation, especially in tropical and semi-arid regions [4,5].

In many situations, crop sowing is performed under inappropriate soil moisture conditions to support seed germination, or in areas with excess salts in the soil or irrigation water [4]. Currently, one-third of the world’s cultivated land, 7% of the total world land, and 50% of the irrigated land are affected by salinity [6]. Therefore, the sustainability of agriculture production in many areas of the world is at risk due to soil salinization and water scarcity during the crop growing season.

Low water content and salt excess in the soil at sowing time cause delayed and reduced seed germination, unequal seedling emergence, and unsatisfactory stand establishment, which results in crop yield reductions [7,8]. Drought and salinity affect seed germination and seedling growth by creating highly negative water potentials, thus preventing water uptake by the seeds and plants [8,9,10,11]. Salinity may also cause direct phytotoxic effects of Na^+^ and Cl^−^ ions [9]. Therefore, drought and salt tolerance testing in the initial stages of plant growth is important because a seed with more rapid germination under water or salt stress conditions may be expected to achieve rapid seedling establishment, resulting in higher yields.

Many factors affect plant responses to drought or salt stress, such as plant genetics, timing, the intensity and duration of applied stress, and environmental factors that determine the genotype versus environment interaction [11,12,13,14]. Genetic differences in tolerance to abiotic stresses in soybean genotypes have been reported in other studies [4,15,16,17], which may be useful in identifying genotypes more adapted to sowing under abiotic stress conditions. In the research by Zuffo et al. [4], the authors proposed a multitrait tool to select the best soybean genotypes exposed to drought and saline stresses. They investigated the stability of 46 soybean genotypes using the stability index. Among the results presented, they mention that this index can be used under different stressful environmental conditions to quantify the genotypic stability of soybean genotypes.

In this research, we discussed how the concept of distance (or similarity) in a vector space could be used to evaluate changes in the characteristics of soybean genotypes when subjected to stressed environments.

Two objects are similar if they have characteristics in common. These objects are represented as vectors in a vector space model (V.S.M.) [18]. Each component is a feature or a characteristic of the object and represents a dimension in the vector space. A real n-dimensional vector *x* (i.e., $x\in {\mathbb{R}}^{n}$) is expressed to its components as $x=(x^{1},x^{2},\ldots ,x^{n})$, where the symbol ℝ represents the real number set. In this text, the features of soybean genotypes are the following variables: germination (GERM), shoot length (SL), root length (RL), total length (TL), shoot dry mass (SDM), root dry mass (RDM) and total dry mass (TDM). Thus, each sample of the dataset is represented as $x\in {\mathbb{R}}^{7}$, that belongs to a 7-dimensional vector space.

In the VSM, the similarity is related to the distance between vectors [19]. In other words, the closer two objects are, the more similar they are. Classic machine learning algorithms, such as a k-nearest neighbor, k-means, support vector machine, and others, use distance metrics to measure similarity [20,21]. There are several ways to calculate the distances between vectors. Some of them are Euclidean, Manhattan, Chebyshev, Mahalanobis, Cosine, Hamming, Jaccard, and Spearman [22].

In this research, the soybean genotype samples are drawn in the VSM. The Manhattan distance is used for compute similarity, as it is more suitable in higher dimensions. It is calculated for different stressful environments. To combine the distance measures and choose which genotype has the shortest distance (higher similarity with the control sample) in both stressed environments, we propose using the Technique for Order Preference by Similarity to Ideal Solution (TOPSIS). This method is a multi-criteria decision-making approach used in several areas [23]. It is preferable to other decision-making approaches because (i) it is suited to a large number of attributes and alternatives; (ii) it requires little subjectivity in the definition of input values; and (iii) it has consistency in the comparison of the alternative ranking [24].

The main objective of this research is to select soybean genotypes exposed to stressful environments (saline or drought) using similarity based on Manhattan distance and the TOPSIS method.

## 2. Results

**Figure 1:**
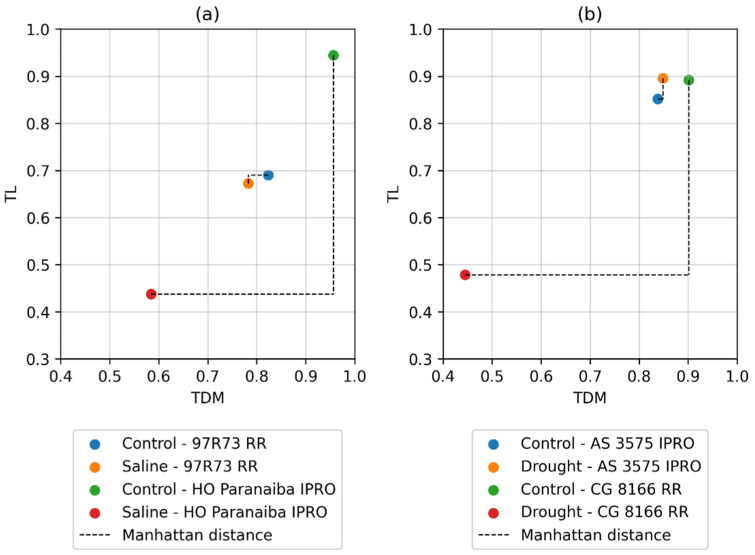
Shows four vectors for only two variables, i.e., TDM (total seedling dry matter) and TL (shoot length). These vectors are related to the most similar (shortest distance) and least similar (greater distance) genotypes regarding the comparison: (**a**) Control/Saline, and (**b**) Control/Drought.

To illustrate the proposed approach, Figure 1 shows a further example considering only two normalized variables, i.e., TDM and TL in Figure 1a, “97R73 RR” and “HO Paranaiba IPRO” are the genotypes with the shortest and greatest distances in the Control/Saline comparison, respectively. In Figure 1b, “AS 3575 IPRO” and “CG 8166 RR” are related to the shortest and greatest distances in the Control/Drought comparison, respectively.

Calculations of Manhattan distances are performed according to Equation (1). Distances were calculated for the Control/Saline and Control/Drought comparison. The estimates are made considering the mean values of four samples of each genotype for the variables GERM, SL, RL, TL, SDM, RDM, and TDM, after the normalization procedure for each variable.

**Figure 2:**
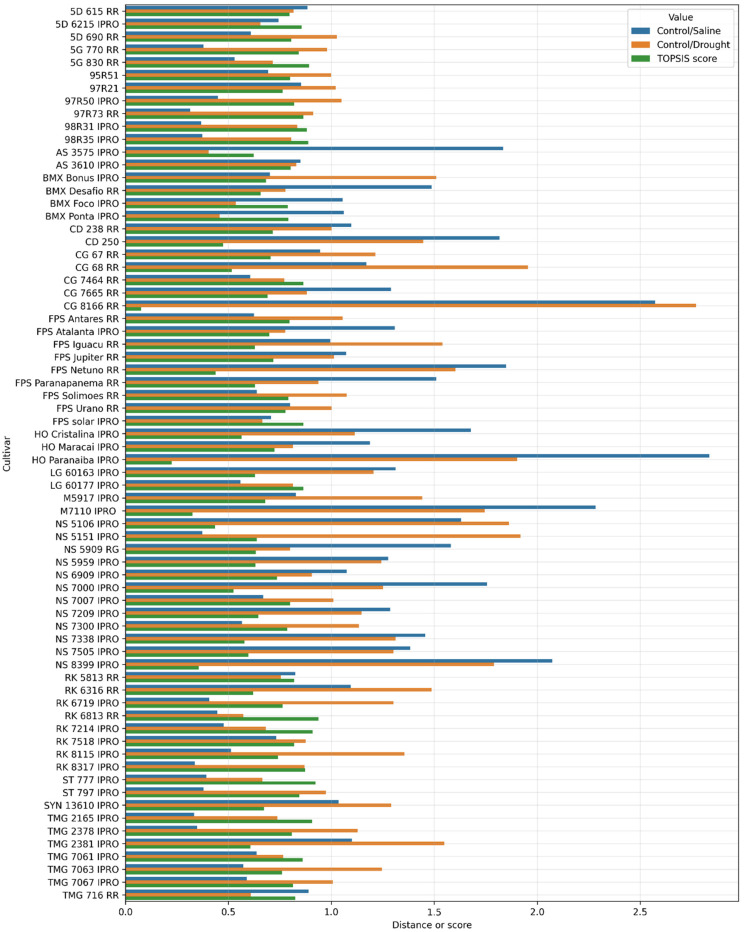
Manhattan distances and Technique for Order Preference by Similarity to Ideal Solution (TOPSIS) score for each soybean genotype.

Figure 2 shows the values of the Manhattan distances obtained in comparing normalized variables between control and abiotic stress environments and the score obtained by the TOPSIS method. For this experiment, the criteria weights (distances) were equal to 0.5. It is noteworthy that the TOPSIS method was employed for the Manhattan distances and not for the variables.

Table 1 shows the ten best soybean genotypes (cultivars) in ascending order, considering the Manhattan distances obtained in the Control/Saline and Control/Drought comparison. The presented results make it clear that no genotype is better than another. Only the genotype ST 777 IPRO appeared on both lists. This trade-off makes the process of choosing the best genotype a difficult task. Therefore, it is necessary to use the TOPSIS method, which has the power to join these distances to decide which genotype performs better in both environments of abiotic stress.

**Figure 3:**
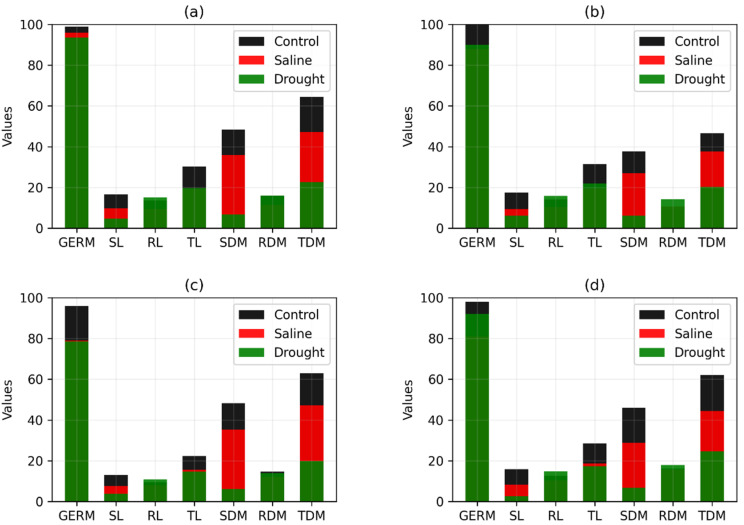
Values of the original variables in the control and abiotic stress environment for the four best soybean genotypes. (**a**–**d**) are the values of genotypes RK 6813 RR, ST 777 IPRO, RK 7214 IPRO, and TMG 2165 IPRO, respectively.

Table 2 shows the ten best genotypes selected according to the TOPSIS score in descending order. In addition, the Manhattan distances of each genotype in each stressed environment are shown, as well as the rank position according to the distances shown in Figure 3.

The genotypes respond differently in each environment of abiotic stress (Table 1 and Table 2). The results shown in Figure 2 assumed the same weight in the TOPSIS method for both Control/Saline and Control/Drought comparisons. To verify how this weighting affects the selection of the genotypes provided by the TOPSIS, the weights of the distances (criteria) are varied from 0.1 to 1, remembering that the sum of the weights is equal to 1, according to Section 4.3. Some results obtained with this experiment are shown in Table 3.

**Figure 4:**
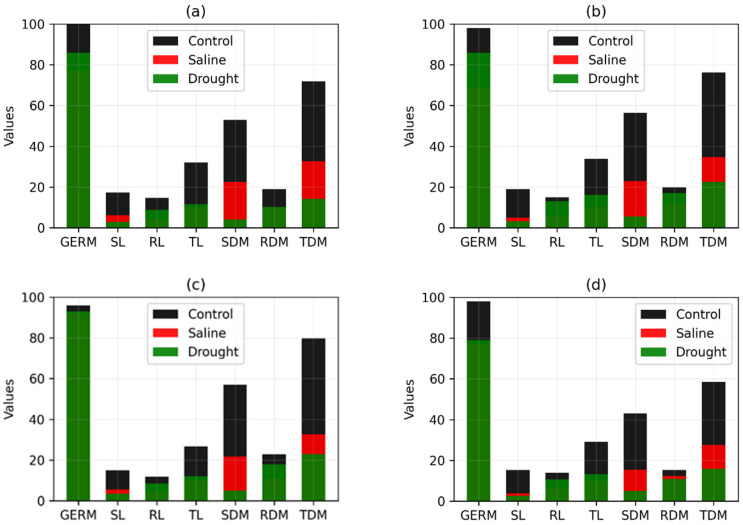
Values of the original variables in the control and abiotic stress environment for the four worst soybean genotypes. (**a**–**d**) are the values of genotypes CG 8166 RR, HO Paranaiba IPRO, M7110 IPRO, and NS 8399 IPRO, respectively.

Finally, Figure 3 and Figure 4 show the original values (without normalization) of the variables GERM, SL, RL, TL, SDM, RDM, and TDM for the four best and worst genotypes, respectively, selected by TOPSIS, considering the same weight for both criteria. This comparison is important to determine if the Manhattan distance and the TOPSIS method are selecting those genotypes that suffer fewer changes in abiotic stress environments.

Table 4 shows the percentage changes (increase or decrease) considering the control and abiotic stress environments. Values are displayed only for the best and worst genotypes according to the TOPSIS selection. The negative sign indicates an increase in the value of the variable.

## 3. Discussion

Our experience reveals that objects with close features are more similar. Distance metrics mathematically verify this notion [19,25]. In this work, it is being investigated whether these metrics can be used to measure the similarity between soybean genotypes in the control environment and abiotic stress environments. For this, it was necessary to model the obtained data in a 7-dimensional vector. We chose the Manhattan distance to calculate the similarity between the samples because it presents better results in high-dimensional vector spaces [18,20,21,22,25]. Since the genotypes show different responses in saline and drought-stressed environments, we also included the TOPSIS method to select the one with the greatest similarity in both environments. Yao et al. [26], using the TOPSIS approach, compared seeds of *Bupleurum chinense* and found that the green ones had a good germination characteristic and were recognized as the superior group, followed by the yellow, brown, and black ones. Successful application of the TOPSIS in dealing with complicated issues in managing crop priority planning has been employed in the soybean crop [27].

As a result of using the Manhattan and TOPSIS distance, Figure 1 illustrates well how distance is related to similarity. The genotype “97R73 RR” (Figure 1a) has the shortest distance in the saline stress environment; therefore, it has greater similarity with the average sample of the control environment. The values of the TDM and TL variables are very close for the samples in the control and saline stress environment. On the other hand, the genotype “HO Paranaiba IPRO” has the greatest distance in the saline stress environment. Therefore, it was observed that, in the stress environment, the values of the TDM and TL variables are very different from those in the control environment. The analogous conclusion can be observed in Figure 1b in the drought-stress environment. In this case, it is important to note that, although the distance was shown only for two variables, the distance metric is generalizable for vector spaces of any dimension [19,25]. The difficulty of selecting genotypes through adaptability studies under abiotic stress conditions such as drought, salinity, and aluminum toxicity has been shown in soybean [3,4,5,14,16], sorghum [6], wheat [7], and corn [11]. Several methods are available to evaluate groups of genotypes in different environments. However, it is still difficult to select the best genotypes because the responses are very variable, so new approaches, such as those described in our work, are important.

The results presented in Figure 2 and Table 1 and Table 2, make it clear that no genotype is absolutely better than another. Some genotypes are more similar to the control samples in the saline stress environment, while others are more similar to the control samples in the drought environment. This is because genotypes respond differently to abiotic stress [3,4,5,14,16]. The genotypes “97R73 RR” and “AS 3575 IPRO” are the most similar in saline and drought stress environments, respectively. However, the genotype “AS 3575 IPRO” occupies the 65th position when considering distance in the Control/Saline comparison. On the other hand, the genotype “97R73 RR” occupies the 28th position in the Control/Drought comparison. The response to different stresses is always variable, as verified in the present work, hence the difficulty in selecting most crops.

Analyzing the results in Table 2, we noticed that none of these genotypes was selected by the TOPSIS method. Moreover, the first genotype selected, i.e., “RK 6813 RR”, occupies the 12th and 4th position concerning the Control/Saline and Control/Drought comparisons, respectively. From the Rank column of Table 2, we conclude that TOPSIS makes a balanced selection of genotypes. If we add the Manhattan distances (4th and 5th columns), we notice that the sum values increase, although the individual values of the distances do not show a different order.

Considering the results in Table 3, which show the selection by TOPSIS when varying the criteria weights (distances), we observed that the genotype “ST 777 IPRO” was selected in all cases. On the other hand, the best genotype, “RK 6813 RR”, was not selected in the last case, when the weights were 0.1 and 0.9 for the saline and drought environments, respectively. These genotypes are less dependent on the weights assigned to distances. In other words, these genotypes present more stability relative to abiotic stress environments and reduced distance values. However, the genotype that presents greater stability, and close similarities to both stress environments, is the genotype “AS 3610 IPRO” (see Figure 2). However, their distances for stress environments are greater than those of the genotypes “RK 6813 RR” and “ST 777 IPRO”. The genotype “AS 3610 IPRO” occupies the 24th position, considering the TOPSIS score. It was shown that the TOPSIS method had practical meaning, confirming the applications made by Li et al. [28].

From the original values (without normalization) of the variables GERM, SL, RL, TL, SDM, RDM, and TDM, it can be seen from the results shown in Figure 3 and Figure 4 that the TOPSIS method made an appropriate selection. The values of these variables for the best (RK 6813 RR) and worst (CG 8166 RR) genotypes, respectively, are shown in Figure 3a and Figure 4a. We noticed that the changes in the values of the variables are much more pronounced for the worst genotype than for the best. In other words, the worst genotype, according to the TOPSIS method, is the one that suffered the most modification in abiotic stress environments. Xue et al. [29] demonstrated that the TOPSIS method could be used efficiently to evaluate the total content of bioactive compounds of different grains, thus providing a database for manufacturing companies to optimally select the germination period.

In Table 4, for example, we note that the variable GERM suffered a decrease of 3.03% and 5.55% in the saline and drought environment, respectively, for the best genotype. On the other hand, for the worst genotype, this decrease was 23% and 14%, respectively. Even more drastic was the change concerning the RL variable. For the best genotype, there was an increase of 10.29%, while for the worst, there was a decrease of over 40%. Similar changes are observed for all other variables, showing that the selection provided by the Manhattan distance combined with the TOPSIS method is correct.

By analyzing the most important strengths and weaknesses of the TOPSIS method, we can say that the method shows the accuracy of results when evaluating a large number of alternatives. Expert knowledge is a basic source of information for making effective managerial decisions in the selection process of plants subjected to abiotic stress.

In addition, a wide variety of techniques have been used to introduce effective factors in the selection of superior genotypes. However, the best or most suitable method is not clearly defined, so various methods are used to ensure the correct decision. The constant evaluation in stress environments combined with selection strategies is constantly sought among soybean breeders to promote the selection of genotypes with the best performance. Therefore, the simultaneous use of methods and the presentation of their most important criteria is the only solution to identifying the best genotypes.

## 4. Materials and Methods

### 4.1. Plant Material and Stress Treatments

Seeds from a total of 70 midwestern Brazilian commercial soybean genotypes [*Glycine max* (L.) Merrill.] listed in Table 5 were produced under field conditions at Cassilândia, MS, Brazil (19°05′16″ S, 51°48′04″ W, and an altitude of 480 m), during the 2019 to 2020 growing season, and used in this study. Minimum and maximum air temperatures during the growing season were 21.7 and 35.3 °C, respectively, and mean air relative humidity ranged from 51 to 83%. The harvest was manually performed at the R8 stage (full maturity), and the plants were air-dried at room temperature for 96 h. The seeds were extracted by hand, sieved through round hole sieves with 6.00 mm diameters, and then stored in sealed paper bags at 13 °C and 35% moisture content until use. Before starting the experiment, the water content, thousand seed weight, and germination rate were determined, as described in the Official Rules for Seed Analysis [30]. The results obtained for the soybean genotypes are shown in Table 5.

The seeds were previously disinfected by immersion for 10 min in 1% sodium hypochlorite solution (*v*/*v*), washed in running water, and placed to germinate under stressful (drought and saline stress) and non-stressful (control) conditions. The drought and saline stresses were induced by exposing seeds from each soybean genotype to solutions with an osmotic potential of −0.20 MPa prepared with polyethylene glycol (PEG-6000) and sodium chloride (NaCl), respectively. The amount of PEG-6000 (119.57 g L^−1^) added to obtain the solution with an osmotic pressure of –0.20 MPa was determined by the equation of Michel and Kaufmann [31]: Ψs = [−(1.18 × 10^−2^) × C − (1.18 × 10^−4^) × C2 + (2.67 × 10^−4^) × C × T + (8.39 × 10^−7^) × C2 × T]/10, where Ψs is the osmotic potential (MPa), C is the concentration (g L^−1^ of PEG-6000), and T is the temperature (°C). The amount of NaCl (2.357 g L^−1^) added to obtain the osmotic pressure of –0.20 MPa was calculated by the van’t Hoff equation [32]: Ψs = −R × T × C × i, where R is the universal constant of noble gas (0.008314 MPa mol^−1^ K^−1^), T is the absolute temperature (273.15 + °C), C is the molar concentration of the solute (mol L^−1^), and i is the van’t Hoff factor, that is the number of ions released when the solute is dissolved in water (i.e., for NaCl this value is 2.0 (Na^+^ and Cl^−^)). Distilled water with an osmotic potential of 0.00 MPa was used as a control. Using an osmotic solution at –0.20 MPa efficiently discriminates the tolerance differences between soybean genotypes [4].

### 4.2. Germination Conditions and Measured Variables

Four replicates of 50 seeds from each soybean genotype were placed to germinate on three sheets of germination test paper towels, previously moistened with distilled water (control), PEG or NaCl solutions of –0.2 MPa, in the proportion of three times the mass of the dry substrate. The paper towel sheets were then turned into rolls and packaged into plastic bags to prevent evaporation and to maintain the relative humidity close to 100%. Germination was conducted in a growth chamber under 12/12 h photoperiod (light/darkness), with a light intensity of 240 μmol m^−2^ s^−1^ and a temperature of 25 °C for 14 days. Seeds were considered germinated when the primary root was longer than 10.0 mm. Germinated seeds were recorded 14 days after the test installation.

After 14 days of exposure to drought and salt stresses, the shoot length (SL), primary root length (RL), and total seedling length (TL) were measured using a meter scale. The shoot dry matter (SDM), root dry matter (RDM), and total seedling dry matter (TDM) were recorded after oven drying at 85 °C for 48 h.

### 4.3. Manhattan Distance and Similarity

The distance function (metric) of two vectors x and y is designed by d(x,y), $d:\;{\mathbb{R}}^{n}\times {\mathbb{R}}^{n}\to \mathbb{R}$, and must satisfy the requirements: (a) d(x,y)>0; (b) d(x,x)=0; (c) d(x,y)=d(y,x); and (d) d(x,y)+d(y,z)≥d(x,z) [18]. The Minkowski distances of two vectors $x=\left(x_{1},x_{2},\ldots ,x_{n}\right)$ and $y=\left(y_{1},\;y_{2},\;\ldots ,\;y_{n}\right)$ are defined as Equation (1):

(1) $$d\left(x,y,p\right)={\left(\sum_{i=1}^{n}{\left|x_{i}-y_{i}\right|}^{p}\right)}^{1/p}$$

**Figure 5:**
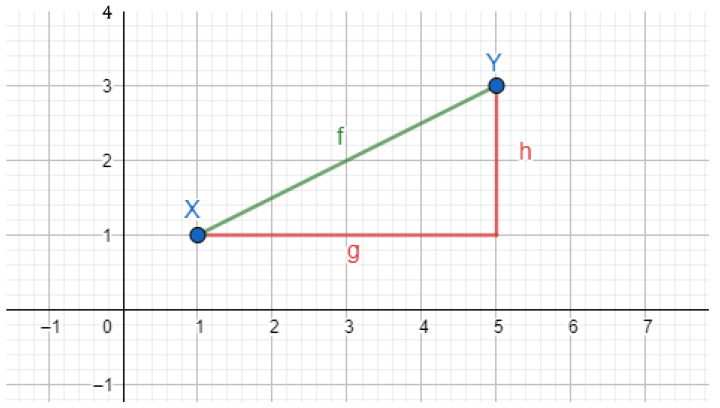
Comparison of Manhattan (g + h) and Euclidean (f) distances between vectors X and Y.

For p=1 and p=2, this distance is named Manhattan and Euclidean, respectively. For comparison, Figure 5 shows these distances in the plane, where d(X,Y,1)=g+h and d(X,Y,2)=f.

Manhattan distance can be understood as the shortest route taken by a taxi driven in a city whose streets are perpendicular. On the other hand, the Euclidean distance is the intuitive notion of distance used by us. Aggarwal, Hinneburg, and Keim [25] establish that the Manhattan distance is the most adequate to contrast the difference between the nearest and farthest vectors from a fixed vector.

Since the variables can be on different scales, applying a pre-processing step named normalization is necessary. This consists of dividing the variables by their maximum value, i.e., ${\tilde{x}}_{i}=x_{i}/\max\left(x_{i}\right)$, where $x_{i}$ is the value of the variable for the i-th sample and ${\tilde{x}}_{i}$ is the respective normalized value. This ensures that ${\tilde{x}}_{i}$ is in a range between 0 and 1. In addition, the scale is eliminated, and the variable becomes dimensionless.

### 4.4. TOPSIS

The TOPSIS method can be employed in six steps, as described below [33]. Let $X={\left(x_{ij}\right)}_{m\times n}$ be a decision matrix with m alternatives and n criteria, where $x_{ij}$ is the value of the alternative i concerning criterion j, do:

Step 1. Normalizes the decision matrix: $r_{ij}=x_{ij}/\overset{m}{\underset{i=1}{\sum }}x_{ij}^{2}\;,\;\forall j$;

Step 2. Given a criteria weight vector $w=\left[w_{1},w_{2},\ldots ,w_{n}\right]$, obtain the weighted normalized decision matrix: $v_{ij}=r_{ij}w_{j}^{T}$, such that $\sum w_{j}=1$;

Step 3. Determine the worst alternative $A_{w}$ (negative-ideal solution) and best alternative $A_{b}$ (positive-ideal solution) as: $A_{wj}=\max_{i=1}^{m}v_{ij}$ and $A_{bj}=\min_{i=1}^{m}v_{ij}$, respectively;

Step 4. Calculate the Euclidean distance from each alternative i to the worst and best alternatives: $S_{i}^{w}=\sqrt{\overset{m}{\underset{i=1}{\sum }}{\left(v_{ij}-A_{wj}\right)}^{2}}$ and $S_{i}^{b}=\sqrt{\overset{m}{\underset{i=1}{\sum }}{\left(v_{ij}-A_{bj}\right)}^{2}}$, respectively;

Step 5. Calculate the relative closeness from each alternative i to the worst alternative: $C_{i}=S_{i}^{w}/\left(S_{i}^{w}+S_{i}^{b}\right)$.

The coefficient $C_{i}$ provides a TOPSIS score to rank the alternatives. The higher its value, the closer that alternative is to the ideal solution. In the context of this study, the alternatives are the genotypes, and the criteria are the Manhattan distances.

### 4.5. Proposed Approach

Our objective is to verify which genotypes are less sensitive to changes in the stressed environment, referring to salinity and drought. As stated in Section 4.3, we can use distance metrics to measure the similarity between objects. The experiments carried out with the soybean genotypes considered three environments: control, saline, and drought. We know that stressed environments alter plant responses. Therefore, there will be changes in the values of the measured variables. We can verify how much these modifications altered the characteristics of the plants. To do so, we can model it as a VSM problem. We calculated the distance between the control samples’ mean and the stressed samples’ mean for each genotype. Then, we compare the distances. Those genotypes that present the smallest distance to the control samples are the genotypes less sensitive to saline and/or drought stress. Our approach considers the Manhattan distance because, according to Aggarwal, Hinneburg, and Keim [25], it is more suitable to contrast the difference between the nearest and farthest vectors from a fixed vector.

A second approach aims to combine distances for the comparison in the stressed environments, that is, Control/Saline or Control/Drought. In this way, we will know which genotypes are more similar to the control samples in both stressed environments. To combine the distances of saline ($d_{s}$) and of drought ($d_{d}$) environments from the control, we propose the use of the TOPSIS method, where the first and second column of the decision matrix is composed of values $d_{s}$ and $d_{d}$, respectively, for each genotype (alternative).

Although TOPSIS can be applied directly in the measured variables, this would not be adequate. Therefore, there is no way to determine whether a greater or lesser value of the magnitude of these variables implies a better or worse genotype. Therefore, it is necessary to use the idea of similarity (or distance). So, we are sure that a smaller distance indicates greater similarity with the control sample.

The entire computational implementation was carried out in the Python language using the Colab environment and the NumPy, Pandas, IPython, Seaborn, Scipy, and Matplotlib libraries [34,35,36,37,38,39,40,41]. The developed source code and experimental data can be accessed at this link: https://github.com/brunobro/selection-of-soybean-genotypes, accessed on 24 August 2022.

## 5. Conclusions

Our results show that the genotypic stability of 70 soybean genotypes can be quantified by the TOPSIS method and Manhattan distance when comparing drought and salinity conditions in relation to the non-stressful environment (control). Based on the TOPSIS method, we can select the best soybean genotypes for environments with multiple abiotic stresses. Among all soybean genotypes tested, RK 6813 RR, ST 777 IPRO, RK 7214 IPRO, TMG 2165 IPRO, 5G 830 RR, 98R35 IPRO, 98R31 IPRO, RK 8317 IPRO, CG 7464 RR, and LG 60,177 IPRO are the 10 most stable under drought and saline stress conditions. Furthermore, from the point of view of plant breeding, these selected genotypes can be used as parents to obtain genotypes resistant to drought and salinity.

## Figures and Tables

**Table 1:** Top ten genotypes to Control/Saline, and Control/Drought distances in ascending order.

Genotype	Control/Saline Distance	Genotype	Control/Drought Distance
97R73 RR	0.3132	AS 3575 IPRO	0.4034
TMG 2165 IPRO	0.3329	BMX Ponta IPRO	0.4578
RK 8317 IPRO	0.3367	BMX Foco IPRO	0.5371
TMG 2378 IPRO	0.3482	RK 6813 RR	0.5733
98R31 IPRO	0.3670	TMG 716 RR	0.6079
98R35 IPRO	0.3720	5D 6215 IPRO	0.6550
NS 5151 IPRO	0.3730	FPS solar IPRO	0.6645
5G 770 RR	0.3787	ST 777 IPRO	0.6649
ST 797 IPRO	0.3800	RK 7214 IPRO	0.6805
ST 777 IPRO	0.3926	5G 830 RR	0.7158

**Table 2:** Top ten genotypes to TOPSIS score in descending order.

Genotype	Rank		Manhattan Distances		TOPSIS Score
	C/S *	C/D **	C/S	C/D	
RK 6813 RR	12th	4th	0.4461	0.5733	0.9380
ST 777 IPRO	10th	8th	0.3926	0.6649	0.9230
RK 7214 IPRO	14th	9th	0.4772	0.6805	0.9081
TMG 2165 IPRO	2nd	11th	0.3329	0.7385	0.9073
5G 830 RR	16th	10th	0.5298	0.7158	0.8911
98R35 IPRO.	6th	18th	0.3720	0.8048	0.8878
98R31 IPRO.	5th	23th	0.3670	0.8342	0.8805
RK 8317 IPRO	3rd	24th	0.3367	0.8696	0.8728
CG 7464 RR	21th	14th	0.6055	0.7714	0.8650
LG 60,177 IPRO	17th	20th	0.5593	0.8136	0.8637

* C/S and ** C/D mean Control/Saline and Control/Draught, respectively.

**Table 3:** Genotypes selected by the TOPSIS when criteria weights (Control/Saline and Control/Drought distances) vary.

Weights		Genotypes	TOPSIS Score
Control/Saline	Control/Drought		
0.1	0.9	BMX Ponta IPRO BMX Foco IPRO AS 3575 IPRO RK 6813 RR TMG 716 RR 5D 6215 IPRO ST 777 IPRO FPS solar IPRO RK 7214 IPRO 5G 830 RR	0.9581 0.9340 0.9315 0.9284 0.9100 0.8925 0.8903 0.8889 0.8836 0.8687
0.3	0.7	RK 6813 RR ST 777 IPRO RK 7214 IPRO FPS solar IPRO 5D 6215 IPRO BMX Ponta IPRO TMG 716 RR 5G 830 RR TMG 2165 IPRO BMX Foco IPRO	0.9313 0.8997 0.8908 0.8802 0.8798 0.8794 0.8768 0.8754 0.8735 0.8700
0.7	0.3	ST 777 IPRO TMG 2165 IPRO RK 6813 RR 98R35 IPRO 98R31 IPRO RK 8317 IPRO RK 7214 IPRO 97R73 RR ST 797 IPRO 5G 770 RR	0.9507 0.9495 0.9444 0.9362 0.9329 0.9307 0.9262 0.9260 0.9125 0.9119
0.9	0.1	TMG 2165 IPRO 97R73 RR RK 8317 IPRO 98R31 IPRO 98R35 IPRO TMG 2378 IPRO ST 777 IPRO 5G 770 RR ST 797 IPRO RK 6719 IPRO	0.9839 0.9788 0.9783 0.9721 0.9713 0.9669 0.9668 0.9647 0.9645 0.9473

**Table 4:** Percentage change of the original variables for the best (RK 6813 RR) and the worst (CG 8166 RR) genotype.

Genotype	Comparison	Percentage Increase/Decrease in Variables						
		GERM	SL	RL	TL	SMD	RDM	TDM
RK 6813 RR	Control/Saline	3.03	40.96	30.69	36.34	25.78	28.72	26.54
	Control/Drought	5.55	71.68	−10.29	34.76	86.30	−0.31	64.90
CG 8166 RR	Control/Saline	23.00	64.84	71.37	67.99	57.50	47.08	54.76
	Control/Drought	14.00	84.14	40.37	64.09	92.31	46.02	80.20

GERM: germination; SL: shoot length; RL: root length; TL: total length; SDM: shoot dry mass; RDM: root dry mass and TDM: total dry mass.

**Table 5:** Agronomic characteristics, seed water content (WC), thousand seed weight (SW), and germination rate (GR) of 70 Brazilian soybean genotypes used in this study.

Genotype	Agronomic Characteristics			WC (%)	SW (g)	GR (%)
	MC	RMG	GT			
5D615 RR	Early	6.1	Ind.	9.97	173	98
5D6215 IPRO.	Early	6.4	Ind.	9.22	156	97
5D690 RR	Early	6.9	Ind.	9.05	176	89
5G 770 RR	Early	7.7	Ind.	9.01	174	100
5G 830 RR	Early	8.3	Ind.	9.67	167	100
95R51	Ultraearly	7.5	Ind.	9.60	177	91
97R21	Early	7.2	Ind.	8.85	176	97
97R50 IPRO.	Early	7.5	Ind.	8.15	190	100
97R73 RR	Mid	7.7	Ind.	9.66	187	97
98R31 I.P.R.O.	Mid	8.3	Ind.	9.89	175	97
98R35 I.P.R.O.	Mid	8.3	Ind.	9.57	180	98
AS 3575 IPRO	Ultraearly	5.7	Ind.	8.84	189	99
AS 3610 IPRO	Ultraearly	6.1	Ind.	9.81	173	98
BMX Bônus IPRO	Mid	7.9	Ind.	9.42	185	100
BMX Desafio RR	Early	7.4	Ind.	8.74	170	99
BMX Foco IPRO	110	7.2	Ind.	9.11	175	83
BMX Ponta IPRO.	Early	6.9	Ind.	9.58	191	98
CD 238 RR	Mid	7.1	Det.	9.43	165	96
CD 250	Mid	5.5	Ind.	9.09	159	100
CG 67 RR.	Mid	7.4	Semi	8.78	165	98
CG 68 RR	Early	6.8	Ind.	9.43	182	86
CG 7464 RR	Early	7.4	Semi.	9.65	159	96
CG 7665 RR	Mid	7.6	Semi.	9.84	192	97
CG 8166 RR	Mid	8.1	Ind.	9.34	174	96
FPS Antares RR	Mid	6.8	Ind.	9.28	194	100
F.P.S. Atalanta I.P.R.O.	Early	5.8	Ind.	9.07	189	88
FPS Iguaçu RR	Ultraearly	5.0	Ind.	9.43	159	100
FPS Júpiter RR	Early	5.9	Ind.	8.59	130	98
FPS Netuno RR	Mid	6.3	Ind.	9.19	135	98
FPS Paranapanema RR	Early	5.6	Semi.	8.43	164	93
F.P.S. Solar I.P.R.O.	Early	6.3	Ind.	8.48	188	99
FPS Solimões RR	Early	5.7	Ind.	9.38	198	97
F.P.S. Urano RR	Early	6.2	Ind.	8.74	278	96
HO Cristalino IPRO	Mid	8.3	Ind.	8.93	160	100
HO Maracaí IPRO	Mid	7.7	Ind.	9.60	170	87
HO Paranaíba IPRO	Early	7.4	Ind.	9.75	210	93
LG 60,163 IPRO	Early	6.3	Semi.	8.95	210	98
LG 60,177 IPRO	Early	7.7	Ind.	9.61	199	99
M5917 IPRO	Ultraearly	5.9	Ind.	9.42	170	93
M7110 IPRO	Early	6.8	Ind.	9.01	195	100
NS 5106 IPRO	Ultraearly	5.2	Ind.	9.11	202	96
NS 5151 IPRO	Ultraearly	5.2	Ind.	9.50	173	95
NS 5909 RG	Ultraearly	6.9	Ind.	9.95	177	80
NS 5959 IPRO	Early	5.9	Ind.	9.18	176	95
NS 6909 IPRO	Ultraearly	6.3	Ind.	8.34	165	92
NS 7000 IPRO	Early	7.0	Ind.	9.40	201	88
NS 7007 IPRO	Early	7.1	Ind.	9.77	210	99
NS 7209 IPRO	Mid	7.3	Ind.	9.34	272	97
NS 7300 IPRO	Early	7.3	Ind.	9.72	190	100
NS 7338 IPRO	Early	7.3	Ind.	9.30	197	94
NS 7505 IPRO	Early	7.5	Ind.	8.97	200	84
NS 8399 IPRO	Mid	8.3	Ind.	9.02	185	89
RK 5813 RR	Ultraearly	5.8	Ind.	8.76	202	98
RK 6316 IPRO	Early	6.3	Ind.	9.51	194	91
RK 6719 IPRO	Early	6.7	Ind.	9.57	190	100
RK 6813 RR	Early	6.8	Ind.	8.30	168	99
RK 7214 IPRO	Early	7.2	Ind.	8.60	178	96
RK 7518 IPRO	Early	7.5	Ind.	10.08	180	100
RK 8115 IPRO	Mid	8.1	Ind.	9.02	200	96
RK 8317 IPRO	Mid	8.3	Ind.	10.46	185	88
ST 777 IPRO	Early	7.7	Ind.	9.41	155	100
ST 797 IPRO	Early	7.9	Ind.	9.45	150	100
SYN 13,610 IPRO	Early	6.1	Ind.	8.53	167	96
TMG 2165 IPRO	Early	6.5	Ind.	9.14	180	98
TMG 2378 IPRO	Mid	7.8	Semi.	8.96	165	96
TMG 2381 IPRO	Mid	8.1	Ind.	9.77	160	100
TMG 7061 IPRO	Early	6.1	Ind.	10.17	185	98
TMG 7063 IPRO	Early	7.0	Ind.	9.75	175	86
TMG 7067 IPRO	Early	7.2	Semi.	9.87	170	100
TMG 716 RR	Early	5.9	Ind.	9.51	167	100

MC: soybean maturity cycle. RMG: relative maturity group. GT: Growth type. Ind.: Indeterminate growing habit. Det.: Determinate growing habit. Semi.: Semideterminate growing habit.

## Data Availability

All available data can be obtained by contacting the corresponding author.
